# High Risk of Metabolic and Adipose Tissue Dysfunctions in Adult Male Progeny, Due to Prenatal and Adulthood Malnutrition Induced by Fructose Rich Diet

**DOI:** 10.3390/nu8030178

**Published:** 2016-03-22

**Authors:** Ana Alzamendi, Guillermina Zubiría, Griselda Moreno, Andrea Portales, Eduardo Spinedi, Andrés Giovambattista

**Affiliations:** 1 IMBICE (CICPBA-CONICET La Plata-National University of La Plata (UNLP)), La Plata 1900, Argentina; aalzamendi@imbice.gov.ar (A.A.); gzubiria@imbice.gov.ar (G.Z.); andreaeportales@gmail.com (A.P.); 2 IIFP (CONICET La Plata) School of Exact Sciences, National University of La Plata (UNLP), La Plata1900, Argentina; gmoreno@iifp.laplata-conicet.gov.ar; 3 CENEXA (CONICET La Plata-UNLP, a PAHO/WHO (Collaborating Centre for Diabetes)), School of Medicine, National University of La Plata (UNLP), La Plata1900, Argentina; spinedi@cenexa.org

**Keywords:** metabolic programming, adipocyte precursor cells, dysfunctional adipose tissue, altered glucose tolerance, insulin resistance, pre-diabetes, metabolic syndrome

## Abstract

The aim of this work was to determine the effect of a fructose rich diet (FRD) consumed by the pregnant mother on the endocrine-metabolic and *in vivo* and *in vitro* adipose tissue (AT) functions of the male offspring in adulthood. At 60 days of age, rats born to FRD-fed mothers (F) showed impaired glucose tolerance after glucose overload and high circulating levels of leptin (LEP). Despite the diminished mass of retroperitoneal AT, this tissue was characterized by enhanced LEP gene expression, and hypertrophic adipocytes secreting *in vitro* larger amounts of LEP. Analyses of stromal vascular fraction composition by flow cytometry revealed a reduced number of adipocyte precursor cells. Additionally, 60 day-old control (C) and F male rats were subjected to control diet (CC and FC animals) or FRD (CF and FF rats) for three weeks. FF animals were heavier and consumed more calories. Their metabolic-endocrine parameters were aggravated; they developed severe hyperglycemia, hypertriglyceridemia, hyperleptinemia and augmented AT mass with hypertrophic adipocytes. Our study highlights that manipulation of maternal diet induced an offspring phenotype mainly imprinted with a severely unhealthy adipogenic process with undesirable endocrine-metabolic consequences, putting them at high risk for developing a diabetic state.

## 1. Introduction

Metabolic Syndrome (MS), obesity and type 2 diabetes have drastically increased to epidemic levels worldwide in the last few decades. The World Health Organization has defined obesity as an increment in adipose tissue (AT) mass which may or may not be detrimental to health [1]. These illnesses have been attributed mainly to changes in lifestyle, including dietary habits. Fructose rich diet (FRD) consumption has been shown to be associated with insulin resistance [2], hyperadiposity, hypertriglyceridemia, hyperleptinemia [3], hyperglycemia, hyperinsulinemia, impaired glucose tolerance [4] and hepatic steatosis [5], in both animal models and humans.

Diseases in adulthood may originate during an individual’s development, due to changes in the environment in which the individual is subjected to [6]. Factors that may alter the environment can include maternal nutrition, either during gestation and lactation [7,8]. Several studies highlighted the undesirable consequences of maternal unbalanced diet intake during gestation and/or lactation for the offspring’s health in adult life [9,10]. Different works have focused on the effects of feeding mothers a FRD during gestation and lactation, showing that their offspring displayed an increased risk of developing obesity in adult life [11,12]. We previously reported deleterious metabolic-endocrine function of pups born to FRD-fed lactating mothers [13]. These pups developed changes in the hypothalamic circuitry controlling appetite, impaired peripheral insulin sensitivity, and both hyperleptinemia and visceral adipocyte hypertrophy [13].

Carbohydrates are major metabolites involved in fetal growth and metabolism, and it is accepted that fructose is present in fetal circulation [14]. The intake of refined sugar, particularly high-fructose corn syrup, has increased in the United States from a yearly estimate of 8.1 kg/individual at the beginning of the 19th century up to 65 kg/person in 2010 [15]. However, limited data exist on the consequences of FRD intake through the mother for the offspring’s metabolic programming [16,17]. Indeed, most of the international literature showed studies wherein experimental designs were devoted to mothers consuming FRD throughout gestation and lactation or only while lactating [11,18,19,20].

It was reported recently that adult male offspring born to FRD-fed dams throughout gestation developed insulin resistance, dyslipidemia, a distorted pattern of peripheral adipokines and enhanced oxidative stress [21]. Interestingly, later in life, the offspring normalized leptinemia, but became hypoadiponectinemic [22]. Conversely, a recent study showed that FRD intake by gestating mothers resulted in pronounced maternal dysfunctions but no major undesirable metabolic effects on offspring, even after following their progress up to 6 months of age [23].

The current study was designed to test whether or not the feeding of mothers with a FRD throughout gestation modifies endocrine-metabolic biomarkers and *in vivo* and *in vitro* AT functions in adult male progeny. Moreover, we aimed to determine *in utero* fructose exposure impact on adult rats challenged or not with a FRD during adult lifetime.

## 2. Materials and Methods

### 2.1. Animals and Experimental Designs

**Figure 1:**
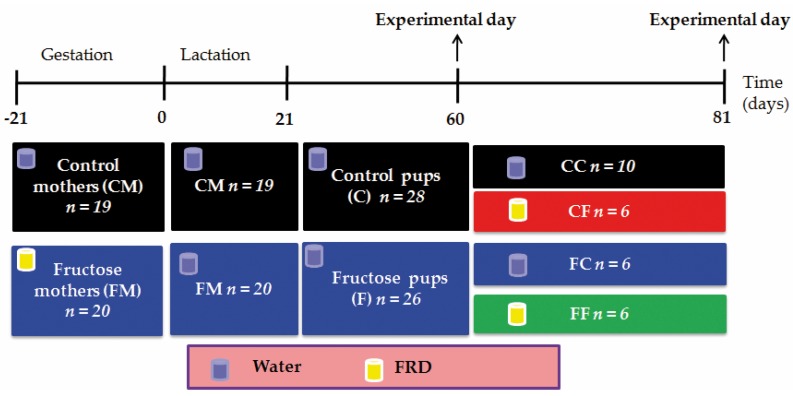
Experimental design. Sperm positive dams fed standard chow *ad libitum* were allocated in two groups: drinking water (control mothers, CM, *n* = 19) and drinking FRD (fructose mothers, FM, *n* = 20). This diet was maintained until delivery. During lactation, both mothers groups were fed standard chow *ad libitum* and drink tap water. The weaning day, male pups born to CM and FM, assigned as C and F rats, respectively, were fed standard purina chow diet and tap water *ad libitum.* At 60 days of age, 6 C and 7 F males were killed for basal determinations and AT studies. Others 6 C and 7 F rats were subjected to an i.v. GTT at 60 day-old. The remaining 16 C rats were divided in two sub-groups; 10 animals receive water (CC) and 6 receive FRD (CF). The remaining 12 F animals, they also were subdivided into two sub-groups; 6 animals receive water (FC) and 6 receive FRD (FF) from day 60 until 81 days of age. Groups were made by including one male rat from each litter, so as not to include pups from the same litter in the same experiment.

Sprague-Dawley (S-D) rats were kept in controlled conditions of temperature (21 ± 2 °C) and lights (on between 07:00 a.m. and 7:00 p.m. with free access to standard commercial rat chow (Ganave Lab., Argentina) and tap water. Virgin females (3 per cage) were mated with a male in plastic cages until positive detection of sperm in their vaginal smears (daily examined at 08:00 a.m.). Sperm positive dams were then individually housed and provided with standard chow *ad libitum* and allocated into two groups: (a) drinking tap water only (control mothers, CM; *n* = 19); and (b) drinking a FRD (fructose 10% w/v in tap water, fructose mothers, FM; *n* = 20) throughout pregnancy [24]. Fresh fructose solution was provided every 2 days. Body weight (BW), food and fluid intakes were recorded daily during pregnancy. Immediately after delivery, and as previously validated by our laboratory [13], litter-size was adjusted to eight pups per dam (average male pups per litter ranged between 60% and 65%, approximately). Lactating mothers were provided with standard chow and water *ad libitum*. Weaned (21 days old) male pups (born to CM and FM, assigned as C and F rats, respectively) were individually housed and fed standard purina chow diet and tap water *ad libitum* until experimentation. These groups of male rats (C and F) were made by including one male rat from each litter (see Figure 1). Rats were euthanized following protocols in the National Institute of Health Guidelines for care and use of experimental animals. All experiments also received approval from our Institutional Committee on Animal Experimentation.

#### 2.1.1. Sixty-Day-Old Rats

Six C and seven F rats were killed (08:00–09:00 a.m.) by decapitation in non-fasting conditions and trunk blood was collected into plastic tubes containing EDTA (10% w/v, in normal saline solution). Plasma samples were then stored frozen (−20 °C) until determination of several metabolites. Immediately after euthanization, retroperitoneal adipose tissue (RPAT) pads were dissected and weighed. RPAT was used for histological, gene expression, stromal vascular fraction (SVF) cell composition and adipocyte functional studies (see below). Additionally, six C and seven F rats were subjected to an intravenous glucose tolerance test (i.v. GTT) (Figure 1).

#### 2.1.2. Eighty-One Day-Old Rats

Sixteen C and 12 F 60-day-old male rats were allocated to four sub-groups according to the drinking solution provided, either tap water only CC (*n* = 10) and FC (*n* = 6) groups; or FRD CF (*n* = 6) and FF (*n* = 6) groups (Figure 1). All groups were fed standard purina chow *ad libitum* and treatments lasted for three weeks. Rat BW and food and fluid intakes were recorded daily. Rats were killed by decapitation (08:00 a.m.) in non-fasting conditions. Trunk blood was collected and plasma samples kept frozen (−20 °C) until measurement of different metabolites. Additionally, RPAT pads were dissected, weighed, and used for histological studies.

### 2.2. RPAT Adipocyte Isolation and Incubation

Isolated adipocytes from RPAT pads were obtained as previously described [25]. Cells were diluted to a density of approximately 200,000 cells per 900 μL of DMEM-1% BSA medium and distributed into 5 mL plastic tubes. Substances tested (diluted in 100 μL) were either medium alone (basal) or contained insulin (INS) (final concentrations: 0.1, 1 or 10 nM; Novo Nordisk Pharma AG, Switzerland) [26]. Adipocytes were then incubated for 45 min at 37 °C in a 95% air-5% CO_2_ atmosphere. At the end of incubation, media were carefully aspirated and kept frozen (−20 °C) until measurement of LEP concentrations as described below.

### 2.3. RPAT Pad Histology

For histological studies, freshly dissected RPAT pads were fixed in 4% paraformaldehyde, then washed with tap water, immersed in a series of graded ethanol (70%, 96% and 100%), and clarified in xylene before being embedded in paraffin [13]. Four-micrometer sections were taken from different levels of the blocks and stained with hematoxylin-eosin. Quantitative morphometric analysis was performed using a RGB CCD Sony camera together with Image Pro-Plus 4.0 software (magnification, ×400). For each tissue sample, seven sections and three levels were selected (*n* = 4 animals per group). Systematic random sampling was used to select 15 fields for each section, and 2500 cells per group were examined. Adipocyte diameter was measured [24] and cell volume was later calculated (4/3π*r*^3^).

### 2.4. RNA Isolation and Real-Time Quantitative PCR

Total RNA was isolated from RPAT pads by the single-step acid guanidinium isothiocyanate-phenol-chloroform extraction method (TRIzol; Invitrogen, LifeTech.). One µg of total RNA was reverse-transcribed using random primers (0.2 µg/µL) and RevertAid Reverse Transcriptase (200 U/µL Thermo Scientific). Primers applied were β-actin (ACTB; sense, 5′-GGTCCACACCCGCCACCAG-3′ and anti-sense, 5′-GGGCCTCGTCGCCCACGTA-3′; 200 bp; Gene Code: NM_031144.3), ADIPOQ (sense, 5′-AATCCTGCCCAGTCATGAAG-3′ and anti-sense, 5′-TCTCCAGGAGTGCCATCTCT-3′; 159 bp; Gene Code: NM_144744.3) and LEP (sense, 5′-GAGACCTCCTCCATCTGCTG-3′ and anti-sense, 5′-CTCAGCATTCAGGGCTAAGG-3′; 192 bp; Gene Code: NM_013076.3). One microliter of the RT mix was amplified with Hot FIREPol EvaGreen qPCR Mix Plus (Solis BioDyne) containing 0.5 μM of each specific primer and using the Light Cycler Detection System (MJMini Opticon, Bio-Rad). PCR efficiency was near 1. Threshold cycles (Ct) were measured in separate tubes by duplicate. Identity and purity of the amplified product were checked by electrophoresis on agarose mini-gels, and the melting curve was analyzed at the end of amplification. Relative changes in the expression levels of one specific gene were calculated by ΔCt analysis [13].

### 2.5. SVF Cell Composition Analysis by Flow Cytometry (FACS)

SVF cells from RPAT pads of C and F animals were isolated and at least 2 × 10^5^ cells (in 100 µL PBS/0.5% BSA) incubated with fluorescent antibodies or respective isotype controls for 1 h at 4 °C. After washing steps, flow cytometry was analyzed using a FACS Calibur flow cytometer (Becton Dickinson Biosciences). A combination of surface cell markers were used to identify adipocyte precursor cells (APCs) as: CD34^+^/CD45^−^/CD31^−^ [27]. Conjugated monoclonal antibodies used were: anti-rat CD34:PE (Santa Cruz Biotechnology Inc., Santa Cruz, CA, USA), anti-rat CD45:FITC (Santa Cruz Biotechnology Inc., Santa Cruz, CA, USA) and anti-rat CD31:FITC (Santa Cruz Biotechnology Inc., Santa Cruz, CA, USA). Samples were analyzed using CellQuest Pro (Becton-Dickinson, San Jose, CA, USA) and FlowJo softwares (TreeStar, San Carlo, CA, USA) [27].

### 2.6. Intravenous Glucose Tolerance Test (i.v. GTT)

This test has been widely validated in our laboratory [13]. Briefly, 60 day-old C and F rats (*n* = 6 C and *n* = 7 F rats) were subjected to an i.v. GTT. Rats were implanted (under light ketamine anesthesia) with an i.v. cannula (in the right jugular vein 48 h before experimentation) kept patent by administering heparin (10 U/mL in sterile saline solution: 100 µL). On the morning of the experimental day (08:00–09:00 a.m.) a small volume of blood was taken in non-fasting condition before (time 0) and 5, 15, 30, 60, and 90 min after i.v. glucose (GLU) (2 g/kg BW; dissolved in sterile saline solution) administration [28]; the blood withdrawn was immediately replaced by a similar volume of sterile artificial plasma. Plasma samples were kept frozen (−20 °C) until determination of GLU and INS concentrations. The area under the curve (AUC) was calculated with GraphPad Prism software using basal (time 0) plasma GLU and INS as baseline.

### 2.7. Peripheral Metabolite Measurements

Circulating levels of GLU, triglycerides (TG) and total cholesterol (TC) were measured by commercial kits (Wiener Lab., Rosario, Argentina). Plasma and medium LEP concentrations [25] and circulating levels of INS [26] and corticosterone (CORT) [29] were determined by specific radioimmunoassays (RIAs) previously validated. LEP (standard curve 0.05–25 ng/mL) coefficients of variation (CV) intra- and inter-assay were 4%–7% and 9%–11%, respectively. INS (standard curve 0.08–10 ng/mL) CV intra- and inter-assay were 3%–7% and 8%–11%, respectively. CORT (standard curve 0.05–50 µg/dL) CV intra- and inter-assay were 4%–6% and 8%–10%, respectively. Plasma levels of adiponectin (ADIPOQ) were measured by commercial ELISA kit following manufacturer’s instructions (Linco Research, Cat. #EZRADP-62 K, standard curve: 3–100 ng/mL CV intra- and inter-assay were 0.43%–1.96% and 4.3%–8.44%, respectively).

### 2.8. Statistical Analysis

Results (expressed as means ± SEM) were analyzed by Student t-test when appropriate. One way ANOVA followed by post hoc comparisons of mean values with the Newman Keuls test, was used to compare different groups. ANOVA with repeated measures was used for analyses of i.v. GTT values. Two-way ANOVA followed by Tukey’s multiple comparison test was used for analyses of the effect of INS on LEP release by adipocytes; and for analyses of male caloric intake from weaning until 60 day-old. The non-parametric Mann-Whitney test was employed to analyze data on tissue mRNA expression [13]. All data was analyzed using the software GraphPad Prism 6.0 on Windows (GraphPad Software Inc., San Diego, CA, USA). *P* values lower than 0.05 were considered statistically significant.

## 3. Results

### 3.1. Pregnant Rats’ Body Weight and Food-Provided Energy Intake

The individual BW of pregnant rats were similar in both groups at the beginning (CM: 249.37 ± 9.83 g, and FM: 244.28 ± 6.16 g; *n* = 19 CM and *n* = 20 FM) and at the end (CM: 366.86 ± 19.04 g, and FM: 366.87 ± 10.11 g; *n* = 19 CM and *n* = 20 FM) of the gestational period. The 21-day average of individual daily energy intake through diet was significantly higher in FM than in CM (84.71 ± 3.41 *vs*. 68.17 ± 5.07 kCal/day/100 g BW, respectively; *p* < 0.05, *n* = 19 CM and *n* = 20 FM).

### 3.2. Body Weight and Caloric Intake in Adult Male Offspring Born to CM and FM

**Figure 2:**
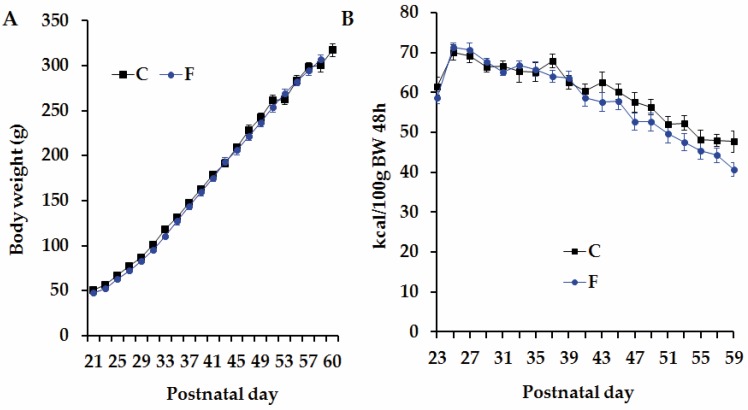
Body weight and caloric intake. (**A**) Body weight and (**B**) mean 48 h cumulative caloric intake corrected by 100 g of BW, between weaning and 60 days of age, in C and F rats. Values are means ± SEM (*n* = 12 and *n* = 14 rats in group C and F, respectively). FRD during gestation, time, and the interaction between them are considered significantly different between C and F rats, based on two-way ANOVA.

The first male offspring born to CM and FM mothers (*n* = 12 and *n* = 14 rats in group C and F, respectively) displayed similar BW between birth and 60 days of age (Figure 2A). No group-difference was observed in 48 h-caloric intake corrected per 100g of BW (Figure 2B). Nevertheless, when a two-way ANOVA analyses was performed, we observed that caloric intake is both influenced by time and FRD intake during gestation was (*p* < 0.05). Even the interaction between these two factors was statistically different (*p* < 0.05).

### 3.3. Peripheral Levels of Several Metabolites and i.v. GTT in Adult Male Offspring

In non-fasting conditions, adult (60 day-old; *n* = 6 C rats and *n* = 7 F rats per group) F rats displayed significantly (*p* < 0.05) higher circulating levels of LEP and lower levels of TG (Table 1). Circulating concentrations of GLU, TC, CORT, INS and ADIPOQ remained the same (Table 1).

**Figure 3:**
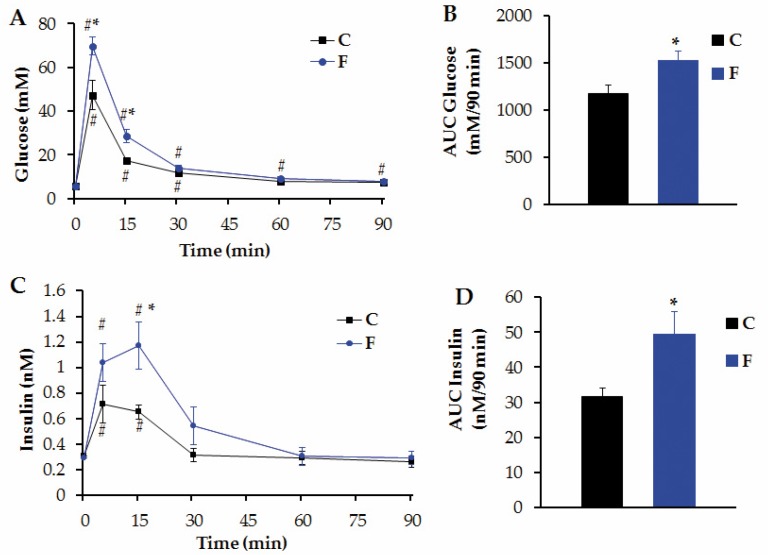
Glucose Tolerance Test. Circulating levels of GLU (**A**), INS (**C**) and the area under the curve (AUC) of GLU (**B**) and INS (**D**) during an i.v. GTT in C and F rats. Values are means ± SEM (*n* = 6 C rats, and *n* = 7 F rats; ^#^ *p* < 0.05 *vs*. time-zero values in the same group, * *p* < 0.05 *vs*. C values in similar conditions). One way ANOVA with repeated measures was used to compare different groups. Student’s *t*-test was used for AUC differences.

Circulating GLU levels (Figure 3A) and the AUC of these peripheral GLU levels (Figure 3B) throughout the i.v. GTT were significantly (*p* < 0.05) higher in F than in C rats (*n* = 6 C and *n* = 7 F rats per group). Moreover, while C rats did recover initial plasma GLU values 60 min after GLU load, F rats failed to do so (Figure 3A). The altered tolerance to GLU overload observed in F rats was related to changes in INS secretion in plasma throughout the test. The profile of circulating levels of INS and the AUC of INS values in both groups are depicted in Figure 3 (C and D, respectively). As shown, plasma INS peak values (time 15 min) were significantly (*p* < 0.05) higher in F than in C rats (Figure 3C). Moreover, we found a significantly (*p* < 0.05) higher AUC for INS values in F than in C rats (Figure 3D).

### 3.4. Retroperitoneal Adipose Tissue Characteristics and Functionality in Adult Male Offspring

**Figure 4:**
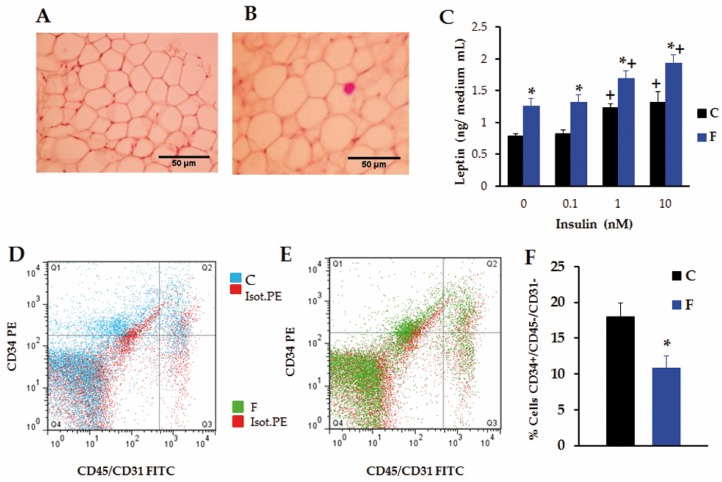
RPAT Characteristics. (**A** and **B**) Histological characteristics, representative fields, in C (**A**) and F (**B**) RPAT pads stained with hematoxylin eosin (scale bar: 50 μm; magnification: ×400); (**C**) Effects of INS on LEP release by adipocytes. Values are means ± SEM (*n* = 4–5 different experiments; * *p* < 0.05 *vs*. C values in similar conditions, ^+^ *p* < 0.05 *vs*. 0 nM INS values in the same group, based on two way ANOVA); (**D** and **E**) Representative dot plots showing the staining profile of RPAT SVF cells isolated from C (**D**) and F (**E**) male adult rats; (**F**) Percentage of CD34^+^/CD45^−^/CD31^−^ cells, determined by flow citometry. * *p* < 0.05 *vs*. C values, based on Student’s *t*-test.

Whereas no difference was found in RPAT mass in 30 day-old C and F rats (data not shown), RPAT fat mass was significantly (*p* < 0.05 *vs.* C) lower in F rats at 60 days of age (Table 2). However, RPAT adipocyte diameter, area and volume were significantly (*p* < 0.05 *vs.* C) higher in F pads (Table 2 and Figure 4A,B, respectively).

Figure 4C shows the results of *in vitro* LEP release by isolated RPAT adipocytes incubated in the absence or presence of graded concentrations of INS. Spontaneous (INS 0 nM) LEP output was significantly (*p* < 0.05) higher in the F than in the C cell-group. While INS 0.1 nM failed to enhance LEP secretion over the baseline in both groups of cells, INS 1 and 10 nM were able to significantly (*p* < 0.05) increase LEP release over the baseline, regardless of the group examined. Interestingly, the amount of LEP released into the medium was significantly (*p* < 0.05) higher in F than in C cell-media, regardless of the condition examined (Figure 4C). These results tally with both circulating levels of LEP (Table 1) and the RPAT LEP mRNA expression levels in these rats (Table 2), although no significant group-difference was noticed in the RPAT mRNA levels of ADIPOQ (Table 2). The adipogenic cell population (CD34^+^/CD31^−^/CD45^−^ cells) in the RPAT SVF from both groups was evaluated in order to assess a possible effect of maternal FRD intake on their APC number (Figure 4D,E). Interestingly, maternal high fructose exposure significantly (*p* < 0.05 *vs.* C) decreased APC number (Figure 4F). This result strongly suggests that the low APC number could be related to the finding of both RPAT adipocyte hypertrophy and decreased pad mass in the F male progeny.

### 3.5. Challenging Adult Male Offspring with a FRD

**Figure 5:**
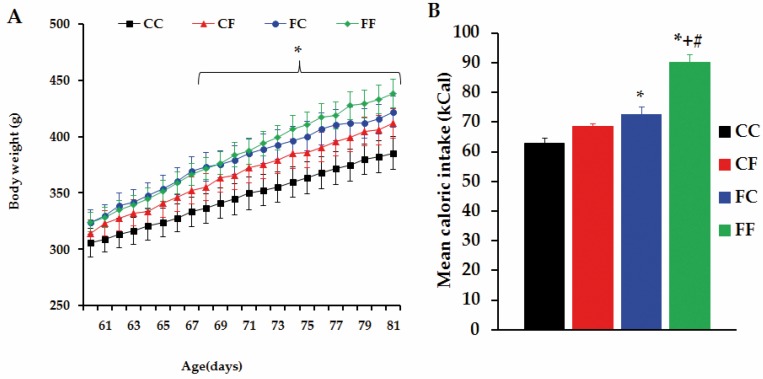
Body weight and mean caloric intake. (**A**) Body weight of male rats, born to CM or FM, fed with either control diet (CC and FC groups) or FRD (CF and FF groups), since postnatal day 60 until day 81; (**B**) Mean caloric intake of animals from four different groups. Values are means ± SEM. *n* = 10 CC rats, *n* = 6 CF rats, *n* = 6 FC rats and *n* = 6 FF rats. * *p* < 0.05 *vs*. CC values, ^+^ *p* < 0.05 *vs*. CF values, ^#^ *p* < 0.05 *vs*. FC values, based on one way ANOVA.

In these experiments, four groups of rats (CC, CF, FC and FF) were studied. The growth curves of rats (studied over a 21 day-period: between 60 and 81 days of age) are shown in Figure 5A. Rat BWs were similar in all groups on the initial day of diet administration. Throughout the indicated period, energy intake (expressed as a 21 day-average) was similar in CC and CF rats. Conversely, this parameter was significantly (*p* < 0.05) higher in FC than in CC rats. Notably, FF rats incorporated the highest (*p* < 0.05 *vs.* remaining group-values) amount of energy (Figure 5B) and, as a result, FF rats were heavier (*p* < 0.05 *vs.* CC rats) from day 68 until 81 days of age, although their final BWs were similar to those from CF and FC rats (Figure 5A). Furthermore, no significant differences were found in final BW values among CC, CF and FC rats (Figure 5A).

When evaluated in basal conditions, CF animals displayed (Table 3) significantly (*p* < 0.05 *vs.* CC values) higher circulating levels of TG, LEP and ADIPOQ; conversely, no difference in plasma GLU and INS levels were noticed. FC rats displayed a severely distorted peripheral profile in several endocrine-metabolic parameters: hyperglycemia, hypertriglyceridemia and hyperleptinemia (*p* < 0.05 *vs.* CC values) although unchanged plasma for INS, TC and CORT (Table 3 data indicating a highly compromised basal metabolic state. Notably, both FC and FF rats showed similar (even *vs*. CC values) basal plasma adiponectinemia, and FF rats were unable (as CF rats did) to mount their adiponectinemia for protection against the FRD challenge applied; moreover, they displayed the highest peripheral levels of GLU, TG and LEP, although with normoinsulinemia (Table 3).

**Figure 6:**
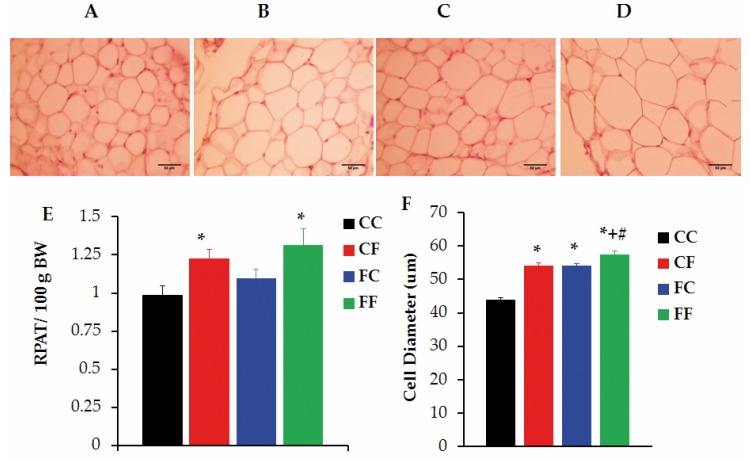
RPAT characteristics. Representative fields of RPAT pads from CC (**A**), CF (**B**), FC (**C**) and FF (**D**) rats stained with hematoxylin eosin (scale bar: 50 μm; magnification: ×400); Additionally, RPAT mass (**E**) and, adipocyte diameter (**F**) from different groups is displayed. Values are means ± SEM. *n* = 10 CC rats, *n* = 6 CF rats, *n* = 6 FC rats and *n* = 6 FF rats. * *p* < 0.05 *vs*. CC values, ^+^ *p* < 0.05 *vs*. CF values; ^#^ *p* < 0.05 *vs*. FF values, based on one-way ANOVA.

Regarding RPAT characteristics, we found that at 81 days-old, pad mass (in g per 100 g BW) was significantly (*p* < 0.05 *vs.* CC values) increased in CF rats and that, although no pad mass difference was observed in FC rats, FF rats also displayed an increased RPAT mass (*p* < 0.05 *vs.* CC values) (Figure 6E). Figure 6A–D displays the morphological characteristics of adipocytes isolated from RPAT pads of different groups. As expected [30], three-week-FRD administration to normal animals (CF group) resulted in enlarged (*p* < 0.05 *vs.* CC) RPAT adipocytes (Figure 6F). Moreover, RPAT pads from rats either challenged (FF) or not (FC) with a FRD at adult age displayed hypertrophic adipocytes (*p* < 0.05 *vs.* CC values), the first being the largest (Figure 6F).

## 4. Discussion

Our results clearly demonstrate a deleterious effect on male offspring’s endocrine-metabolic and adiposity functions induced by feeding pregnant rats a FRD. The consequences of this diet intake by the pregnant mother were evident when pups reached 60 days of age. At that time, male offspring showed dyslipidemia, hyperleptinemia, impaired glucose tolerance and adipocyte hypertrophy; several dysfunctions were further aggravated after offspring were re-challenged with FRD for three weeks.

Epidemiological and experimental studies demonstrated a relationship between maternal nutrition and long-term metabolic consequences in the offspring. Under-/malnourishment [31,32,33] or over-nourishment [34,35] in pregnant mothers induces in their offspring severe endocrine, metabolic and adiposity dysfunctions. In effect, FRD intake during both pre- and early post-natal periods impairs INS and LEP cell signaling, thereby modifying carbohydrate metabolism in the progeny [11,16,19]. Previous studies have shown that mothers consuming a FRD during both gestation and lactation resulted in offspring characterized by dyslipidemia [36] and insulin resistance [37]. Other studies showed that offspring consumption of a rich carbohydrate-milk during lactation increased plasma levels of INS and LEP, and also BW, resulting in pancreatic disorders [12,32,33]. Moreover, FRD-intake by pregnant rats was reported to affect both mother and fetal metabolism by enhancing lipogenesis and hepatic endoplasmic reticulum stress [38]. The deleterious consequences of FRD administration to pregnant mothers have been previously addressed. This diet is able to induce altered glucose tolerance, hyperinsulinemia and reduced placental vascular area, thus leading to high risk for the mother of developing gestational diabetes and preeclampsia [24]; moreover, their fetuses (embryonic day 20) displayed increased BW. Interestingly, these dysfunctions were fully prevented by metformin co-treatment [24], thereby indicating that impaired overall insulin sensitivity in the mother seems to be mainly responsible for these FRD effects.

A relevant observation made throughout the present study is that the detrimental consequences on AT endocrine function seen in F animals (which never consume FRD) are similar to those developed by CF animals. In both situations, a higher basal leptinemia and hypertrophic adipocytes from RPAT were found. However, while FRD administration to the adult offspring is able to enhance adiponectinemia in CF animals, we found that in F animals there was no change in plasma adiponectin concentration. These findings highlight that the AT seems to be the main target for this diet-noxa, and highlight the importance of the perinatal environment for an individual’s development.

It is well known that AT endocrine dysfunction associated to obesity is closely related to adipocyte size, rather than to pad mass [39], and hypertrophic adipocytes are characterized by impaired insulin sensitivity [40] and changes in the adipokine secretory pattern, including higher leptin production [39,41]. Therefore, enhanced adipocyte size could well explain the hyperleptinemia found in F animals despite AT mass decrease. Changes in LEP concentration could contribute to impaired peripheral insulin sensitivity [42]. In the present study, the male offspring born to FRD-fed gestating mothers displayed an apparent paradoxical situation; although, they have a lower AT mass it has large adipocytes. These results could indicate a lower number of adipocytes. It has been found that, in both humans and animal models, AT development occurs mainly during late pregnancy and early postnatal life [43,44,45]. In fact, the ability to generate new adipocytes in adult life is limited; as a consequence, the number of adipocytes remains relatively stable. Therefore, it is possible to speculate that lower adipocyte numbers is a consequence of the alterations in APC determination during the AT development, leading to a decrease in adipocyte generation [43]. Then, considering this unusual situation, we next examined the cellular composition of the RPAT SVF to determine whether cell hypertrophy and low pad mass could be a result of a lower APC number. Interestingly, a low number of APCs, CD34^+^/CD45^−^/CD31^−^ [46] was found in F RPAT pads. This diminished APC number could be responsible for an impaired adipogenic potential [45], and therefore leading to the development of hypertrophic adipocytes. To our knowledge, this is the first study that shows changes in APC numbers induced by excessive prenatal fructose intake.

Indeed, malnutrition during perinatal life could trigger changes in DNA methylation and in histone acetylation/methylation, causing transcriptional changes of key factors involved in adipogenesis (C/EBPα, PPARγ) [47,48]. Examples of this emerge from several studies, such as those findings indicating that, during differentiation of 3T3-L1 cells, there is a modification in the DNA methylation degree (*i.e.*, PPAR, C/EBP, LEP, GULT4) [49,50,51,52] and in histone methylation/acetylation [53], thus demonstrating the relevance of epigenetic regulation during the adipogenic process. It is reasonable to speculate that, at least partially, AT dysfunction found in offspring born from FRD mothers could be a consequence of epigenetic changes. Further research is needed to better clarify the role of epigenetic modifications in our model. Given the endocrine-metabolic alterations that we found in 60 day-old animals from FRD-fed mothers, we proposed studying the response of these rats to a direct challenge with FRD in adult life. For this purpose, we treated C and F 60 day-old rats with a FRD or a normal diet for three weeks (achieving 81 days of life), which has been widely used as an animal model of the human MS [30,54]. When older (81-day-old) pups were studied, surprisingly, we found a remarkably deteriorated AT function and metabolic state in FC pups. These rats had higher basal plasma levels of TG and GLU than CC rats, despite no changes in insulinemia, whereas offspring’s hyperleptinemia and RPAT hypertrophic adipocytes remained. In this regard, it has been previously reported that FRD intake in rats induced severe basal hyperglycemia, with concomitant normoinsulinemia, accompanied by a high risk of cardiovascular events [55]. In our experiments with 81 day-old rats, we noticed that the FRD challenge to control animals (CF), increased circulating levels of TG, LEP and ADIPOQ, as well as RPAT pad mass and adipocyte size, confirming our previous data [30]. Nevertheless, the consequences of direct FRD administration to male offspring born to FRD-fed mothers (FF rats) were even more serious: they showed increased caloric intake, body weight, and plasma levels of GLU, TG and LEP, thus indicating they could be developing diabetes type 2.

Unlike the FC group and similar to findings in the 60-day-old F male offspring, there was no increase in peripheral levels of ADIPOQ. It is well known that ADIPOQ acts as an insulin-sensitizing factor [56]. In fact, ADIPOQ regulates glucose metabolism by improving insulin signaling pathway [57], enhancing liver and muscle glucose uptake, decreasing hepatic glucose output [58,59] and enhancing fatty acid oxidation [60]. Therefore, an increase in ADIPOQ plasma levels observed in 81 day-old control animals challenged with FRD (CF rats in Table 3) might play a protective role in carbohydrate metabolism. These results are in full agreement with those previously reported from our laboratory [30]. The lack of a physiological increase in adiponectin secretion in FF animals is highly indicative of the loss of its protective effect on carbohydrate dys-metabolism, contributing to the worsening of overall metabolic derangements.

## 5. Conclusions

Overall, the present study shows that maternal consumption of fructose during gestation alters offspring’s development causing metabolic and AT dysfunctions. At 60 days of age, rats born to FRD-fed mothers showed impaired insulin sensitivity and profound AT dysfunction, evidenced by hypertrophic adipocytes that secrete *in vitro* larger amounts of LEP, although with decreased AT mass. This paradoxical situation could be the result, at least partially, of the reduced APC number, present in RPAT from F rats. At 81 days old, the results of FC rats corroborate that the impact of FRD on pups worsened the metabolic profile throughout their lifetime, even when they were not directly exposed to this diet. These changes are clearly aggravated when the offspring directly consume FRD during adulthood.

Considering that hypertrophic expansion of AT mass is a key marker for AT dysfunction, we further conclude that high fructose consumption by pregnant mothers primes the first male generation with a high risk of developing MS, obesity and type 2 diabetes. To our knowledge, this study is the first to report changes in adipose precursor cells numbers, induced by *in utero* diet manipulation. However, the impact of high fructose *in utero* on overall developmental programming of individuals requires deeper research.

## Figures and Tables

**Table 1:** Metabolic markers in 60 day-old rats.

	C	F
GLU (mmol/L)	6.58 ± 0.18	6.08 ± 0.24
TG (mmol/L)	1.44 ± 0.14	1.12 ± 0.06 *
TC (mmol/L)	1.56 ± 0.07	1.88 ± 0.13
CORT (nmol/L)	4.67 ± 1.08	3.43 ± 1.02
INS (nmol/L)	0.31 ± 0.02	0.26 ± 0.05
LEP (ng/mL)	2.41 ± 0.35	4.10 ± 0.31 *
ADIPOQ (μg/mL)	5.87 ± 0.72	5.60 ± 0.44

Circulating levels (in non-fasting conditions) of several metabolic markers in 60 day-old male rats born to either normal diet (C)- or FRD (F)-fed mothers throughout pregnancy. Values are means ± SEM. *n* = 6 C rats and *n* = 7 F rats. * *p* < 0.05 *vs*. C values, based on Student’s *t*-test.

**Table 2:** RPAT pad characteristics in 60 day-old rats.

	C	F
**Pad mass (g per 100g BW)**	0.71 ± 0.07	0.54 ± 0.03 *
**Adipocyte diameter (µm)**	37.21 ± 0.09	48.45 ± 0.28 *
**Adipocyte area (µm^2^)**	1182.34 ± 10.64	2057.79 ± 23.57 *
**Adipocyte volume (μm^3^ × 10^3^)**	26.97 ± 1.84	59.97 ± 3.47 *
**LEP mRNA (AU)**	1.09 ± 0.19	2.99 ± 0.18 *
**ADIPOQ mRNA (AU)**	1.28 ± 0.40	1.94 ± 0.41

RPAT pad characteristics in 60 day-old male rats born to either control diet- (C) or FRD-fed (F) mothers throughout pregnancy. Values are means ± SEM. *n* = 6 C rats and *n* = 7 F rats. * *p* < 0.05 *vs*. C values; based on Student’s *t*-test. mRNA expression levels were analyze by the non-parametric Mann-Whitney test.

**Table 3:** Metabolic markers in 81 day-old rats.

	CC	CF	FC	FF
GLU (mmol/L)	4.96 ± 0.27	5.1 ± 0.33	5.88 ± 0.28 *	7.91± 0.56 *^,+,#^
TG (mmol/L)	1.19 ± 0.16	1.89 ± 0.17 *	1.7 ± 0.12 *	2.62 ± 0.11 *^,+,#^
TC (mmol/L)	0.26 ± 0.03	0.23 ± 0.01	0.23 ± 0.001	0.21 ± 0.02
CORT (nmol/L)	140.81 ± 37.41	87.45 ± 24.15	84.52 ± 12.99	190.98 ± 58.14
INS (nmol/L)	0.28 ± 0.04	0.31 ± 0.04	0.37 ± 0.06	0.34 ± 0.05
LEP (ng/mL)	2.87 ± 0.14	3.89 ± 0.38 *	3.89 ± 0.43 *	5.57 ± 1.4 *
ADIPOQ (μg/mL)	14.82 ± 0.94	20.08 ± 2.77 *	13.61 ± 1.95	14.89 ± 2.72

Circulating levels (in non-fasting conditions) of plasma metabolites in 81 day-old male rats from different groups. Values are means ± SEM. *n* = 10 CC rats, *n* = 6 CF rats, *n* = 6 FC rats and *n* = 6 FF rats.* *p* < 0.05 *vs*. CC values, ^+^ *p* < 0.05 *vs*. CF values, ^#^ *p* < 0.05 *vs*. FC values, based on one-way ANOVA.
